# Intubation exposure associated with first-pass success – understanding practitioner-related characteristics in South African EMS

**DOI:** 10.1186/s12245-026-01183-4

**Published:** 2026-03-17

**Authors:** Jaimen Brown, Naseef Abdullah, Simpiwe Sobuwa

**Affiliations:** 1 Department of Emergency Medical Sciences, Faculty of Health & Wellness Sciences, Cape Peninsula University of Technology, Cape Town, South Africa; 2 Emergency Medical Services, Western Cape Government Health and Wellness, Cape Town, South Africa; 3 Department of Emergency Medical Care & Rescue, Faculty of Health Sciences, Durban University of Technology, Durban, South Africa

**Keywords:** Emergency medical services, Endotracheal intubation, First-pass success, South Africa

## Abstract

**Background:**

Endotracheal intubation is a critical life-saving intervention in prehospital emergency care, yet evidence supporting its safe implementation by prehospital practitioners in lower- to middle-income countries remains limited. First pass success (FPS) rates serve as a key process quality indicator, potentially influenced by practitioner-specific factors including clinical experience and procedure exposure. Differentiation between these two distinct measures is critical in understanding their impact on FPS.

**Objective:**

To describe the association between practitioner experience and intubation procedure exposure, and first-pass success rates during prehospital endotracheal intubation.

**Methods:**

We conducted a retrospective review of all prehospital intubations performed by practitioners of a public Emergency Medical Service in South Africa between January 2022 and December 2023. Data were extracted from the EMS case registry, which included integrated Computer Aided Dispatch and electronic Patient Care Record systems. Multivariate logistic regression analysis was used to assess associations, with mixed-model logistic regression identifying practitioner variability.

**Results:**

Among 530,371 primary case activations, a total of 236 intubations were performed by 40 prehospital practitioners during the study period. The overall FPS rate was 80.9% (95% CI: 75.9–86.0%). Practitioner experience ranged from 0 to 14 years (mean 5.7 ± 3.5 years), with procedure exposure ranging from one to 55 intubations (mean 5.9 ± 11.2 intubations). Procedure exposure showed a statistically significant positive association with FPS (OR = 1.020, 95% CI: 1.000–1.040, *p* < 0.05). However, clinical experience showed no statistically significant association with FPS (OR = 0.940, 95% CI: 0.850–1.030, *p* = 0.190), while practitioners with zero to three years of experience demonstrated one of the highest FPS rates (83.33%).

**Conclusion:**

This study reveals a significant association between intubation procedure exposure and first-pass success rates in prehospital intubations, while practitioner experience demonstrated a non-significant association. These findings highlight the importance of continuous professional development in South African prehospital airway management and reinforce the imperative for sustained commitment to clinical excellence as a driver of system growth and improvement.

**Graphical Abstract:**

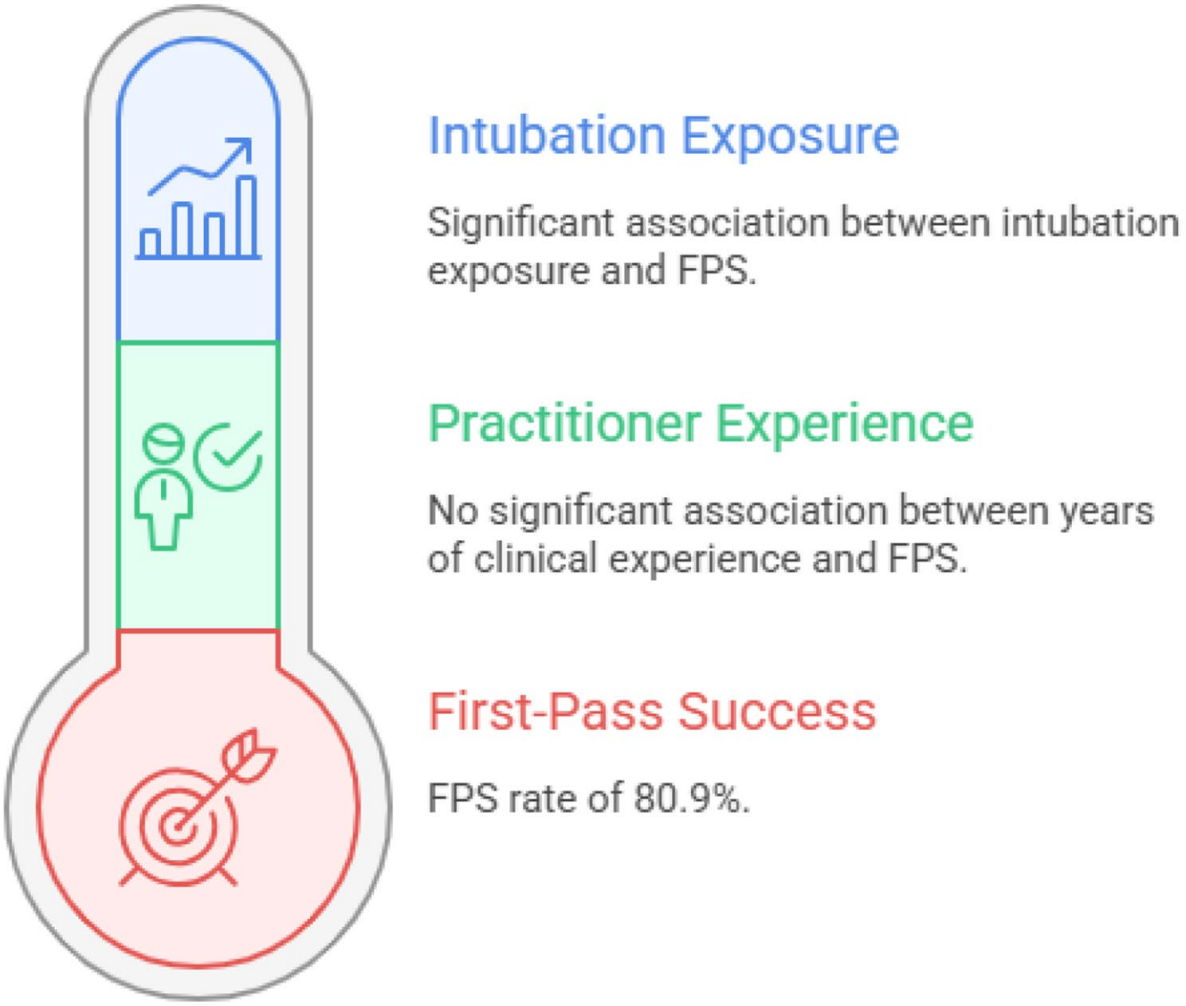

## Background

Endotracheal intubation is widely recognised as the preferred modality for definitive airway management in emergency care, particularly in the prehospital setting where environmental challenges and patient acuity converge to create complex clinical scenarios [1–3]. First-pass success (FPS) has emerged as a broadly accepted process quality indicator for intubation, defined as successful endotracheal tube placement on the first laryngoscopy attempt [4]. FPS in the prehospital setting has not been associated with mortality [5] and its correlation with patient outcomes has been questioned [6, 7]. However, it remains a valuable process metric that reflects technical proficiency and procedure execution, and is thus used as a proxy measure for intubation quality, while being simple and feasible to obtain and determine [5]. While the 2025 European Resuscitation Council guidelines describe a high intubation success rate of over 95% within two intubation attempts during cardiac arrest [8], no universally accepted benchmark for an optimal FPS rate has been established [9]. FPS rates in emergency department (ED) setting are reported at approximately 84.1% [4, 9, 10], while prehospital rates vary considerably, ranging from 59% to 98% [11, 12]. More recent evidence suggests improving prehospital FPS rates, often exceeding 90% [11, 13], signalling an intentional shift towards clinical excellence within emergency medical services (EMS).

Despite its recognised importance, the practitioner-centric factors underpinning successful intubation, particularly practitioner experience and procedure exposure, remain poorly understood in the prehospital context. Prehospital airway management systems are heterogeneous worldwide, with some contexts – particularly within Europe – where intubations are performed almost exclusively by physicians [14]. In paramedic-led systems, such as, Australia, New Zealand and South Africa, advanced airway management interventions are performed by prehospital practitioners [15]. This structural variability influences both the volume of intubation opportunities and the pathways for maintaining procedure competence, underscoring the need to understand how practitioner factors affect performance. Yet the current literature provides limited guidance in this regard.

The preponderance of airway-related literature originates from high-income country (HIC) EDs and conflates years of clinical experience with procedural competency, overlooking the distinct contributions of actual procedure exposure and ongoing skill maintenance [16–18]. Definitions of intubation competency also vary considerably, with recommendations ranging from 35 to 200 intubations for initial physician competency [19–21]. Beyond this, evidence defining the minimum frequency required to maintain skill proficiency is inconsistent. The ED data suggest that three intubations annually may be sufficient [22], while some prehospital guidelines recommend two intubations per month [23]. There is a paucity of literature describing individual practitioner intubation frequencies in prehospital systems, and ED data varies significantly, averaging at three to ten intubations annually [22, 24]. This highlights the absence of well-defined minimum competency standards and contemporary practices expectations.

Against this backdrop, the contribution of practitioner experience and procedure exposure to FPS rates in prehospital settings remains poorly defined, particularly in low- and middle-income countries (LMICs) where paramedic-led airway management predominates. Recent evidence from HICs suggests that higher rates of intubation attempts are associated with improved overall and first-pass success [25]. Building on this, the present study examines the relationship between practitioner experience, intubation procedure exposure, and FPS performance within a South African prehospital system. By doing so, it aims to generate empirical evidence to inform minimum competency expectations, guide workforce development, and support system-level quality improvement.

## Methods

### Study design and setting

We conducted a retrospective descriptive analysis of all prehospital intubations performed by the Western Cape Government Health and Wellness Emergency Medical Services (WCGHW EMS) from January 2022 to December 2023. This research follows the STROBE reporting standards. The South African health system is based on a tiered model, with community-based primary facilities referring patients to secondary and tertiary facilities as needed [26]. Within this context, EMS provides a dual function: primary case activation, whereby the patient enters the health system via EMS from the community, or interfacility transfers [27]. In both settings, there is no specialist or physician oversight, either at the roadside or during interfacility transfers. Consequently, EMS practitioners independently provide care across the full spectrum of practice, from basic to advanced interventions [27, 28]. Prolonged transport times to facilities, particularly in rural areas, and overburdened facilities further delay definitive care [29, 30], reinforcing the critical role of EMS within the health system [27].

The Western Cape province is situated at the southernmost tip of Africa and is home to 7.4 million residents across diverse urban and rural environments. The WCGHW EMS manages approximately 600,000 annual activations [31]. The South African EMS system operates under a three-tier qualification structure: entry-level prehospital practitioner (one-year training), mid-level prehospital practitioner (two- to three-year Diploma), and professional qualification holder - Emergency Care Practitioner (four-year bachelor’s degree) [32]. The latter register with the Health Professions Council of South Africa (HPCSA) as Emergency Care Practitioners (ECPs) and are the only practitioners authorised to perform endotracheal intubation [33]. As detailed in Table 1, only 1.2% of the South African prehospital workforce are categorised as ECPs, with the vast majority qualified as entry-level practitioners [32, 34]. For every intervention they are credentialed to perform, including intubation, ECP graduates are deemed competent through practical simulation and supervised practice during their undergraduate years [35]. Following this, no internship program exists for these practitioners before independent registration as an ECP [32]. Importantly, the South African EMS system is non-physician led, with intubations performed primarily by ECPs using rapid sequence intubation [15].

**Table 1:** Emergency care qualifications framework [34]

	Prehospital Qualifications	Licensed to perform intubation	Proportion of National Prehospital workforce
Tier 1 - Entry-Level	Basics Ambulance Assistant (BAA)	No	94.2%
	Ambulance Emergency Assistance (AEA)	No	
	Emergency Care Assistant (ECA)	No	
Tier 2 - Mid-Level	Emergency Care Technician (ECT)	No	4.6%
	Paramedic (ANT^a^)	No	
Tier 3 - Professional Qualification	Emergency Care Practitioner (ECP)	Yes	1.2%

^*a*^*ANT* Ambulans Nood Tegnikus

When a patient requires intubation, the first ECP to arrive typically performs the procedure and assumes overall responsibility for patient management. Equipment used for such management is standardised across the WCGHW EMS. This includes adjuncts such as bougies and stylets [36], end-tidal carbon dioxide monitoring devices [37], and mechanical ventilators, with no availability of video laryngoscopes [38].

### Study population and sampling

All intubations performed by WCGHW EMS practitioners during the study period were eligible for inclusion. We utilised two integrated data sources: Computer-Aided Dispatch (CAD) systems that capture operational data, and electronic Patient Care Records (ePCR) that document clinical interventions.

Data were extracted from the WCGHW EMS case record registry. The data included all intubations performed outside of a health care facility by non-physician practitioners operating within the WCGHW EMS. The study excluded any intubations performed by EMS practitioners within health care facilities, by physicians, or by practitioners not operating within the WCGHW EMS. Paediatric patients younger than five years were excluded from the analysis. The age of five years is recognised internationally as a key developmental and clinical threshold, reflected in global child health indicators [39]. Intubation strategies also differ in young paediatric populations compared with older paediatrics and adults [40]. Furthermore, a limited number of intubations were recorded in the under-five-year subgroup, and such a small subsample is unlikely to provide stable or meaningful inferences and could compromise the robustness of the overall analysis [41].

### Data collection

Data extraction was performed systematically by a single investigator (J.B.). All prehospital practitioners’ names were cross-referenced with the HPCSA public register to verify credentials and determine registration dates as ECPs. Names were subsequently encoded to ensure anonymity.

Quality assurance measures included double-entry verification for 10% of cases and systematic identification of missing variables by the second author. Cases with incomplete attempt documentation or unverifiable practitioner information were excluded and documented.

### Variables

Intubation attempts were defined as distinct laryngoscopy procedures, each documented numerically by the performing practitioner within the ePCR system. First pass success was defined as the correct placement of the endotracheal tube requiring only a single attempt. Experience was defined as years since registration as an ECP with the HPCSA, up to the beginning of the study period (January 2023).

### Statistical analysis

Descriptive statistics were calculated for all variables, with categorical data presented as frequencies and percentages, and continuous data as means with standard deviations. For inferential analysis, a multivariate logistic regression model was used. The model adjusted for exposure, experience, airway difficulty, intubation method (RSI versus non-RSI), patient type (trauma versus medical), and time of day (day versus nighttime). Further mixed-model logistic regression analysis was performed to analyse FPS variability between practitioners.

We calculated 95% confidence intervals for all estimates and used *p* < 0.05 as the threshold for statistical significance. All analyses were conducted using SPSS version 26.0 (IBM Corp., Armonk, NY).

## Results

**Fig. 1:**
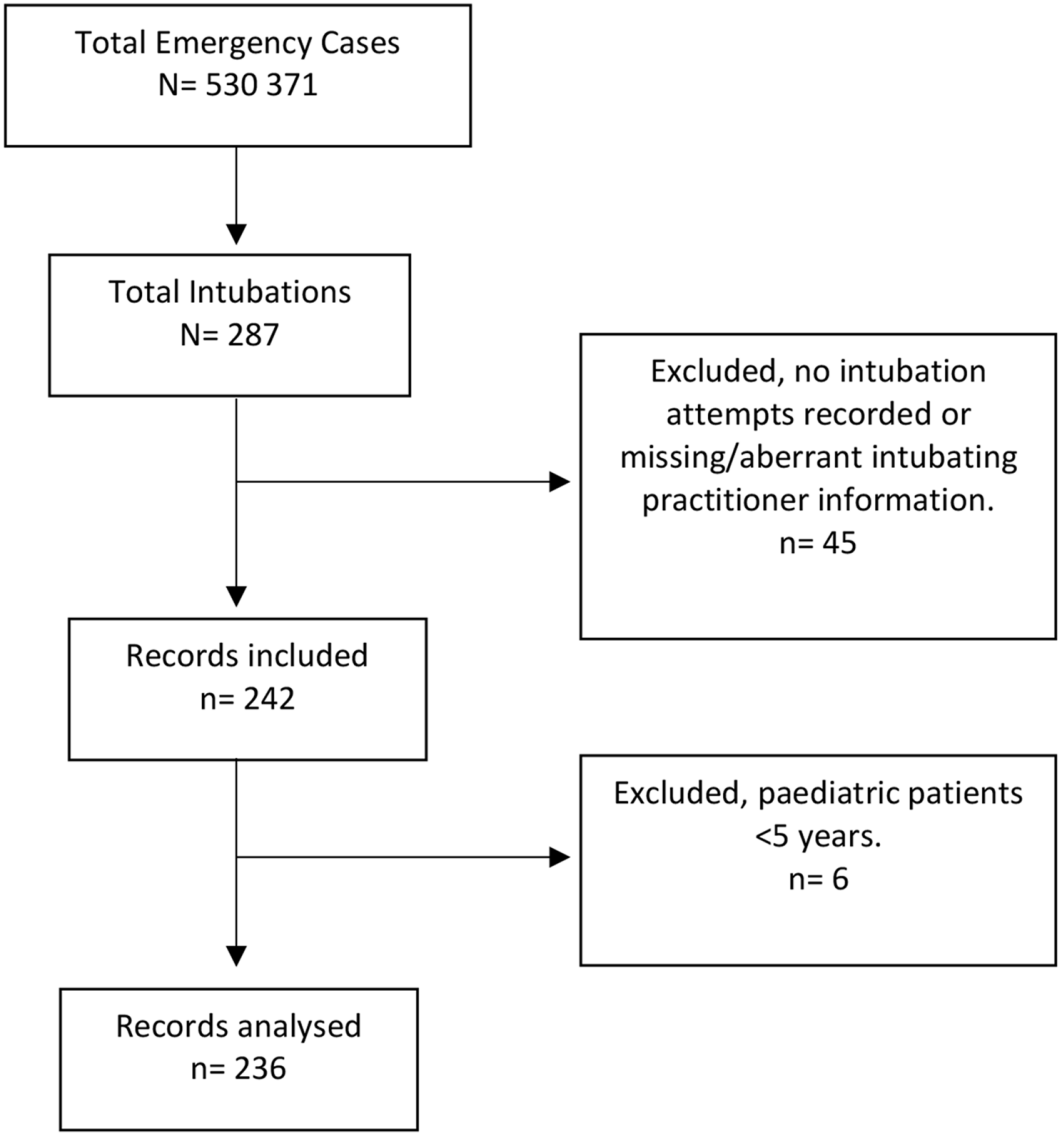
Diagram of final study sample

During the study period, 530,371 primary case activations were recorded, of which 287 (0.05%) patients required intubation (Fig. 1). The remaining activations included patients who either did not require advanced airway management or were not managed with intubation despite clinical indication. After applying the exclusion criteria, 236 cases performed by 40 prehospital practitioners were included in the final analysis.

Table 2 indicates the descriptive statistics summary. Practitioners had a median procedural exposure of 17 intubations, with an interquartile range of 43, indicating a wide range of experience across this cohort. Practitioners had a median experience of 6 years. 191 intubations were recorded as being a first-pass success, thus, the overall FPS rate was 80.9% (95% CI: 75.9–86.0%). Most cases were performed using the RSI method (84.7%), while 82.2% of cases involved trauma patients. 38.1% of cases were documented as difficult airways. Intubations occurred more frequently during the day (58.5%) compared to at night (41.5%).

**Table 2:** Descriptive statistics

Summary	
Practitioners (N)	40
Exposure, number of intubations*	17.00 (5.00; 48.00)
Experience, years*	6.00 (2.00; 11.00)
First-pass success, n (%)	Yes: 191 (80.9%)
	No: 45 (19.1%)
RSI, n (%)	Yes: 200 (84.7%)
	No: 36 (15.3%)
Difficult airway, n (%)	Yes: 90 (38.1%)
	No: 146 (61.9%)
Trauma, n (%)	Yes: 194 (82.2%)
	No: 42 (17.8%)
Time of intubation, n (%)	Day: 138 (58.5%)
	Night: 98 (41.5%)

*Median (25th; 75th percentile)

Table 3 summarises the experience of the 40 prehospital practitioners analysed in this study. Practitioners’ experience ranged from zero to 14 years (mean 5.7 ± 3.5 years). The majority of practitioners had six to seven years of experience as ECPs (*n* = 13, 32.5%). The highest FPS was observed in practitioners with eight to nine years of experience (*n* = 3, 100%). Counterintuitively, practitioners with zero to three years of experience demonstrated one of the highest FPS rates (*n* = 11, 83.33%).

**Table 3:** Practitioner experience and mean FPS^a^ rates

Experience* (0–14 years)	Practitioners (*n* = 40 (%))	Mean FPS** (%)
0–1 years	5 (12.5)	83.33 (± 17.99)
2–3 years	6 (15.0)	83.33 (± 12.37)
4–5 years	8 (20.0)	71.43 (± 22.57)
6–7 years	13 (32.5)	75.51 (± 23.49)
8–9 years	3 (7.5)	100 (± 0)
10–11 years	2 (5.0)	85.51 (± 15.82)
12–14 years	3 (7.5)	62.5 (± 36.32)

^a^*FPS* First Pass Success

*Number of years since registration

** The mean FPS and standard deviation stipulated, is that of each of the stratified groups within the model

Table 4 summarises procedure exposure, which ranged from one to 55 intubations per practitioner (mean 5.91 ± 11.2). Most practitioners (*n* = 32, 80%) performed fewer than six intubations during the study period. A distinct trend in the reduction of standard deviation is observed with increasing exposure, particularly among practitioners with ≥ 6 intubations. This indicates improved reliability and consistency between practitioners with greater exposure. However, the limited sample size and the post hoc nature of the analysis preclude meaningful statistical inference.

**Table 4:** Practitioner exposure and mean FPS^a^ rates

Exposure* (1–55 intubations)	Practitioners (*n* = 40 (%)	Mean FPS** (%)
1 intubation	15	60.00 (± 28.24)
2–3 intubations	10	75.00 (± 24.36)
4–5 intubations	7	81.48 (± 22.68)
6–10 intubations	4	81.25 (± 17.74)
11–20 intubations	2	87.10 (± 17.77)
> 20 intubations	2	83.49 (± 12.66)

^a^*FPS* First Pass Success

*Number of intubations

**The mean FPS and standard deviation stipulated, is that of each of the stratified groups within the model

Table 5 presents results from the multivariate logistic regression model, adjusted for clinically relevant predictors. Exposure emerged as a significant predictor of FPS (*p* < 0.05), with each additional intubation indicating a 2% increased likelihood of achieving FPS. Difficult airway was the strongest predictor of FPS in the adjusted model (*p* < 0.001), wits presence indicating a 90% greater likelihood of first-pass failure. Nighttime also demonstrated significance (*p* = 0.050), indicating a two-fold greater likelihood of first-pass failure. In contrast, experience (*p* = 0.190), RSI (*p* = 0.790) and trauma (*p* = 0.340) were not associated with FPS.

Mixed-model logistic regression revealed no detectable variability between practitioners. This suggests that FPS rates were consistent across practitioners after adjusting for incident and practitioner characteristics.

**Table 5:** Multivariate logistic regression for first-pass success

	Adjusted OR^a^	95% Confidence Interval		*p*-value
		Lower	Upper	
Exposure (per intubation)	1.020	1.000	1.040	0.032
Experience (years)	0.940	0.850	1.030	0.190
RSI^a^ (Yes*)	1.140	0.400	3.030	0.790
Difficult Airway (Yes*)	0.096	0.040	0.210	< 0.001
Trauma (Yes*)	1.600	0.590	4.190	0.340
Time (Day*)	0.470	0.220	1.000	0.050

^a^OR Odds Ratio ^b^RSI Rapid Sequence Induction

*Reference

## Limitations

Several limitations must be acknowledged. The retrospective design and reliance on secondary data may have introduced limitations in data completeness and detail, despite rigorous data cleaning and inclusion criteria. The exclusion of 45 cases (19%) due to incomplete data represents a meaningful loss of information and may have introduced selection bias. Focusing on a single public EMS may limit generalisability to other settings with different training standards, protocols, or patient populations. The two-year study period may not reflect longer-term trends in practitioner performance or practice evolution. Some variables that may influence FPS were not captured in the dataset, including the reason for intubation and aggravating conditions. Patient outcome was not measured, limiting the ability to assess the clinical significance of the observed FPS rates. Two practitioners accounted for 106 intubations, substantially more than their peers. These outliers both operate within specialised clinical units and likely experienced greater intubation exposure than the average practitioner. Finally, the exclusion of neonatal and paediatric patients observed limit generalisability across patient populations and necessitate future research into these patient groups.

## Discussion

Despite the recognised importance of airway management in prehospital care, few studies have explored practitioner-level factors influencing first-pass success (FPS), particularly in low- and middle-income countries (LMICs) and non-physician-led Emergency Medical Service (EMS) systems. The majority of airway management-related literature originates from emergency departments in high-income countries (HICs). In these settings, clinical experience is often conflated with procedural competence, limiting exploration of the independent impact of actual procedural exposure. This study contributes new evidence to this under-researched area.

This study identified an overall FPS rate of 80.9%, with a higher FPS rate among novice practitioners, although experience showed no statistically significant association with FPS. In contrast, procedure exposure did demonstrate a statistically significant association with FPS.

The observed overall FPS rate of 80.9% represents an improvement over previous South African studies and falls within the range reported in similar literature [4, 15, 42, 43]. The modest improvement may reflect the impact of the 2018 prehospital clinical practice guideline revisions mandating RSI. However, the absence of further gains suggests limited clinical growth and a lack of sustained quality-improvement initiatives.

The absence of an association between years of clinical experience and FPS aligns with international literature questioning the value of clinical seniority as a proxy for procedural competency [1, 11]. This is further supported by the high FPS observed among practitioners with the least experience. The strong performance observed among novice practitioners may reflect contemporary undergraduate training programs, where structured supervision and current RSI protocols facilitate skill acquisition [44]. This finding aligns with education literature suggesting that structured, competency-based training may be more effective than experiential learning alone for technical procedures [45, 46]. However, this finding raises concerns about skill maintenance post-graduation, as FPS rates appear to decline with increasing experience unless supported by ongoing training and exposure. This underscores the need for ongoing education, regular skills maintenance and clearly defined competency benchmarks.

The observed relationship between exposure and FPS echoes international findings [11, 16, 18]. Greater procedural exposure improves familiarity with airway management, reduces cognitive load, and enhances technical execution, ultimately leading to higher FPS [46, 47]. Furthermore, the observed relationship between exposure and variability suggests that increasing procedural exposure may be associated with lower between-practitioner variance in FPS, indicating improvements in intubation consistency. This pattern is less variable among practitioners with ≥ 6 intubations over the study period, although group sizes were small. Given that this threshold was derived post hoc from exploratory analysis, it should be considered hypothesis-generating and requires prospective validation before informing competency or credentialing standards. Nonetheless, it is broadly consistent with emergency department literature suggesting that procedural volume relates to performance [22]. This underscores the importance of ongoing skills maintenance of the intubation procedure through routine performance [22], and may offer a novel, evidence-based reference point for both clinical education and workforce planning. This finding further supports several recommendations for prehospital airway management programs that incorporate continued training and education, quality assurance, competency standards and a rethink of the current system design.

These findings highlight the need for structured, system-level clinical improvement initiatives that focus on sustained skill maintenance rather than on initial training or sporadic clinical exposure. Skill maintenance programs should integrate simulation-based training, structured clinical exposure and quality assurance processes that monitor individual procedure volumes.

Future studies should employ prospective designs with standardised data collection to improve data quality and enable more sophisticated analyses. Longitudinal studies tracking individual practitioners over extended periods would provide insights into skill acquisition and maintenance patterns. Further investigation into airway management in neonatal and paediatric patient groups is required to determine competency benchmarks of these populations. Additionally, research into the total number of intubations performed over a practitioner’s lifetime would further assist in developing competency benchmarks. Finally, studies evaluating different training modalities and competency maintenance strategies would inform optimal educational approaches.

## Conclusion

This study suggests that procedure exposure, rather than clinical experience, is a key determinant of FPS in a non-physician-led EMS system. Systems should ensure minimum exposure thresholds while implementing supportive measures for practitioners with limited procedure volumes.

These findings highlight the importance of continued professional development and the need to develop an exposure-driven competency framework. However, achieving and maintaining clinical excellence in prehospital intubation requires a multifaceted approach combining high-quality training, ongoing education, and systematic quality assurance. These principles may extend beyond intubation to other high-stakes, low-frequency procedures in prehospital care.

## Data Availability

Public access to the dataset is closed. Administrative permission in the form of IRE approval was required to access the raw data. The datasets used and analysed are available from the corresponding author on reasonable request.
